# Genetic transformation and growth index determination of the *Larix olgensis LoHDZ2* transcription factor gene in tobacco

**DOI:** 10.1038/s41598-021-99533-0

**Published:** 2021-10-20

**Authors:** Peiqi An, Chen Wang, Qing Cao, Qingrong Zhao, Ruofan Qin, Lei Zhang, Hanguo Zhang

**Affiliations:** 1 State Key Laboratory of Tree Genetics and Breeding (Northeast Forestry University), Harbin, 150040 China; 2 Chinese Academy of Forestry, Beijing, 100000 China

**Keywords:** Biological techniques, Genetics, Molecular biology

## Abstract

Homeodomain-leucine zippers (HD-Zip) are plant-specific transcription factors that participate in different plant development processes and differentially regulate metabolic processes. *LoHDZ2* is an HD-ZipII subfamily transcription factor gene that we identified from a transcriptomic analysis of *Larix olgensis*. To understand its function, we built a *LoHDZ2* expression vector and then inserted it into tobacco by genetic transformation. Transgenic plants were identified at the DNA and RNA levels. Phenotypic index analysis of transgenic tobacco showed dwarfed growth with larger leaves and earlier flowering than the wild type. *LoHDZ2* was expressed differently after hormone treatment with IAA, MeJA and 2,4-D. The results suggested that *LoHDZ2* may respond to hormones and be involved in regulating growth and metabolism. These results helped us better understand the function of *LoHDZ2* and provided a candidate gene for *Larix olgensis* molecular breeding.

## Introduction

The development and growth of plants is controlled by genes, the external environment and internal physiology^1^. Unsuitable Environmental conditions for the growth of plant usually result in stress. Stress in plants is mainly divided into biotic stress and abiotic stress. Abiotic stresses during plant growth, such as salt stress, drought stress, and cold stress, are universal in nature^2^.

HD-Zip transcription factors are plant-specific transcription factors. They belong to the homeobox protein family and have a highly conserved HD (homeodomain)^3^. Based on the characteristics of sequence conservation, genetic structure and physiological function, HD-Zip transcription factors can be separated four subfamilies, such as HD-ZipI, HD-ZipII, HD-ZipIII and HD-ZipIV^4^. Different subfamilies have differences in their genetic composition and the structure of their encoded proteins. Therefore, these transcription factors participate in different plant development processes and differentially regulate metabolic processes. According to previous study, Tril (trichome-less) encodes an HD-ZipIV transcription factor plays a significant role in the primary growth of trichomes and fruit spines^5^. HD-ZipII transcription factors may regulate plant development through growth protein pathways^6^.Overexpression of ECHB1 inhibited defoliation and thus improved growth of eucalyptus under drought stress, reducing water loss from trees by reducing leaf area, but not changing stomatal morphology^7^. HD-ZipII transcription factors affect rice plant development through modulating gibberellin biosynthesis^8^.

*Larix olgensis* is an important coniferous species in China. It has a straight, high-quality and durable trunk^9^. In this study, 50 mg · L^−1^ GA_3_, IAA, ABA, 6-BA, MeJA or 2,4-D solutions or water (control) was sprayed on larch seedlings prior to extracting RNA, which was reverse transcribed into cDNA. Using qRT-PCR to analyzed the expression level of *LoHDZ2*. Additionally, the coding region of *LoHDZ2* was cloned using the leaf-disk method and *Agrobacterium,* resulting in *LoHDZ2* overexpression in transgenic tobacco. Afterwards, the phenotypic changes were measured, and the expression level of *LoHDZ2* in transgenic tobacco under different treatments was analyzed. According to the above research, *LoHDZ2* plays a role in the growth of *Larix olgensis*.

## Materials and methods

### Plant materials and growth conditions

Plant materials are collected and cultivated in accordance with relevant national and international legislation and guidelines, and the cultivation method is carried out under the guidance of professors.

Full and glossy *Larix olgensis* seeds were selected and then sown in a vegetative soil substrate with a 5:3:2 ratio of clear soil:vermiculite:perlite. The conditions were 8 h dark culture and 16 h light culture, 75% humidity, and the temperature is 22 °C.

The tobacco used in this experiment came from the Forest Genetics and Breeding Laboratory of Northeast Forestry University. Collect and use in accordance with relevant national and international regulations and guidelines. First, the seeds were soaked in 20% sodium hypochlorite for 15–20 min, washed with aseptic water 6–7 times, and then inoculated on MS solid medium to germinate, such that sterile seedlings were obtained^10^. Tissue culture seedlings with good growth were selected for propagation as receptor materials for genetic transformation.

### Quantitative real-time PCR analysis

Young larch seedlings that had been growing healthy and strong for 30 days were transplanted into a seedling pot and cultured in a nutrient soil matrix for 2–3 months. Then, larch seedlings were sprayed with 50 mg · L^−1^ GA_3_, IAA, ABA, 6-BA, MeJA or 2,4-D solution or water (as a control), and whole larch seedlings were collected after treatment at 0 h, 2 h, 4 h, 8 h, 12 h, 24 h, 48 h, 72 h and 96 h^11^. RNA was extracted and reverse transcribed into cDNA for qRT-PCR.

Primer5 software was used to design quantitative primers (*LoHDZ2-RT-F:* CTTGGCGTTGGTGTGTCTATG; *LoHDZ2-RT-R*: TGGGCATGAACCAAAGAAAC). Using an ABI7500 fluorescent qRT-PCR instrument, the dissolution curve was determined according to standard procedures, with the following reaction procedure: 94 °C for 30 s, followed by 40–45 cycles of 94 °C for 5 s, 58 °C for 15 s, and 72 °C for 10 s. The differences in the three CT values were all less than 1. Microsoft Excel 2016 was used for data analysis, and GraphPad Prism5 software was used for graphing; the internal reference gene of *Larix olgensis* is MF278617 on NCBI. The expression of *LoHDZ2* under different treatments was analyzed based on the qRT-PCR results.

### Plasmid construction

According to the reference *LoHDZ2* (gene number is MW206675 on NCBI) gene sequence, specific primers were designed to amplify vector fragments: *LoHDZ2-*BamHI-F: GGATCCATGGAAGAGATGAAGAACAAGCA; *LoHDZ2-*Xbal-R: TCTAGATTAGCAAGCTGCAGACTGTTGG. The PCR system contained 1 µl of template, 1.5 µl each of the upstream and downstream primers, 25 µl of KOD FX PCR buffer, 10 µl of dNTPs, 1 µl of KOD FX, and ddH2O to a final volume of 50 µl. The reaction conditions were as follows: 94 °C for 2 min; 40 cycles of 98 °C for 30 s and 68 °C for 1.5 min; and 68 °C for 10 min.

**Figure 1:**
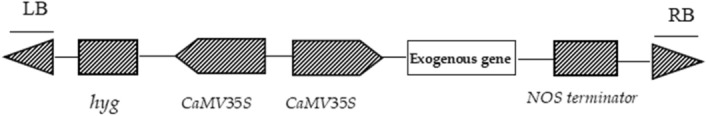
Diagram of the pCAMBIA1302 overexpression vector.

After the reaction, the PCR products were subjected to agarose gel electrophoresis, and recovery of target fragment by a gel recovery kit. The restriction enzymes BamHI and Xbal were used to insert the product into the plant expression vector pCAMBIA1302 (Fig. 1). Using T4 ligase to connect the product and vector., then the vectors were inserted into *E. coli* DH5α cells that were then grown on LB medium containing kanamycin at 37 °C for 12 h to 16 h. PCR was performed to confirm vector uptake by the bacteria.

The positive transformants were sent to Kumei Biotechnology Company for sequencing, and the sequencing reaction was preserved for plasmid extraction. The plasmid p1302*-LoHDZ2* was transferred into *Agrobacterium tumefaciens* GV3101 competent cells by the liquid nitrogen freeze–thaw method, and PCR was performed for detection, with preservation of the bacterial solution.

### Transient expression of GFP in tobacco by injection

Based on the designed primer sequence of *LoHDZ2* CDs sequence, the enzyme digestion sites of Xbal and Spel required by VB191104-eGFP vector were added, and the termination codon TAA at the 3 'end of the downstream primer was removed: *LoHDZ2-Xbal*-F: TCTAGAATGGAAGAGATGAAGAACAAGCA; *LoHDZ2-*Spel-R: ACTAGTAGCAAGCTGCAGACTGTTGG. The plasmid of p1302-*LoHDZ2* was used as template and VB191104-eGFP-*LoHDZ2*-S/A was used as primer for PCR amplification. The PCR product was detected by electrophoresis with 1.2% agarose gel. Then recovered the target strip and the gel. The product was named VB191104-*LoHDZ2*-eGFP.

Then the restriction enzymes Xbal and Spel were used to insert the product into the plant expression vector VB191104-eGFP, then T4 ligation was performed and transformed into *E. coli* DH5α cells competent state,the method is the same as “Plasmid construction”. The positive transformants were sent to Kumei Biotechnology Company for sequencing, and the sequencing reaction was preserved for plasmid extraction. The plasmid VB191104-*LoHDZ2*-eGFP was transferred into *Agrobacterium tumefaciens* GV3101 competent cells by the liquid nitrogen freeze–thaw method.

We injected into tobacco leaves with Agrobacterium VB191104-*LoHDZ2*-eGFP as control 2 ~ 5 days after injection, GFP fluorescence signal was detected under portable long wavelength UV lamp, and GFP signal was detected by fluorescence microscope or laser copolymerization fluorescence microscope.

### Antibiotic susceptibility test

As the overexpression vector used in this study was a modified vector from our laboratory, it was necessary to screen the tobacco plants for sensitivity to hyg. Hygromycin can produce an obvious phenotype in a relatively short amount of time at low concentrations and has a large degree of toxicity to the recipient material, so screening is easily performed^12^.

In this study, wild-type tobacco seeds were selected and disinfected with a 20% bleach solution in water and then washed 5–6 times with sterile water. Seeds were inoculated on 1/2 MS growth medium containing 0, 15, 20, 25, 30 and 35 mg · L^−1^ hyg for one week; then, their growth was observed, and the optimal concentration of hyg was selected.

### Genetic transformation and verification of transgenic plants

The genetic transformation of tobacco was carried out according to the leaf-disk genetic transformation system^13^. After 23 d of coculture in medium with 25 mg · L^−1^ hygromycin and 250 mg · L^−1^ cephalosporin, cultivation was performed for approximately 3 weeks to induce stem growth, and seedlings were transferred to rooting medium. Extracted the DNA of resistant plants’ leaves and detected using the pCAMBIA1302 vector primers via PCR. The PCR product was subjected to 1.2% agarose gel electrophoresis to screen for positive T_1_ seeds_._ The seeds of the T_1_ generation were cultured on the resistance medium and planted in the vegetative soil matrix after seed germination, and after flowering, plants from the T_2_ generation were determine to be transgenic plants.

The total RNA of different transgenic, T_2_ generation tobacco lines and wild tobacco was extracted after 30 days, and reverse transcribed cDNA was used as a template. Then, qRT-PCR was performed by using quantitative gene primers. The detection and calculation methods were the same as in Sect. 2.2.

### Growth index

Twenty samples of the OE2, OE4 and OE5 transgenic plants and wild-type tobacco were propagated and transplanted to nutritional soil matrix. The plants were cultured under long days in an artificial climate culture chamber, and photographs of transgenic and wild-type tobacco at different time periods (in transplanting to soil at 30 d, 60 d and 90 d) were collected to measure plant height, leaf width and leaf length based at least 20 seedlings. Microsoft Excel 2016 statistical software was used to analyze the data, and GraphPad Prism 5 software was used to generate figures.

The above experimental analysis included at least three biological replicates. At the same time, calculating the change of plant height, leaf height and leaf width of the three transgenic lines and wild-type tobacco, the formula (a-b)/a*100% (a: plant height in the previous period; b: plant height in the later period) was used^14^.

### Determination of physiological indexes under hormone treatment

A solution containing 50 mg·L^−1^ IAA, MeJA and 2,4-D was sprayed on plants from three transgenic lines and wild-type tobacco, and three mixed samples were selected from each line after treatment for 0 h, 12 h, 24 h, 48 h and 96 h. The total RNA of the mixed samples was extracted and reverse-transcribed into cDNA for qRT-PCR.

## Results

### qRT-PCR of LoHDZ2 under different hormone treatments

**Figure 2:**
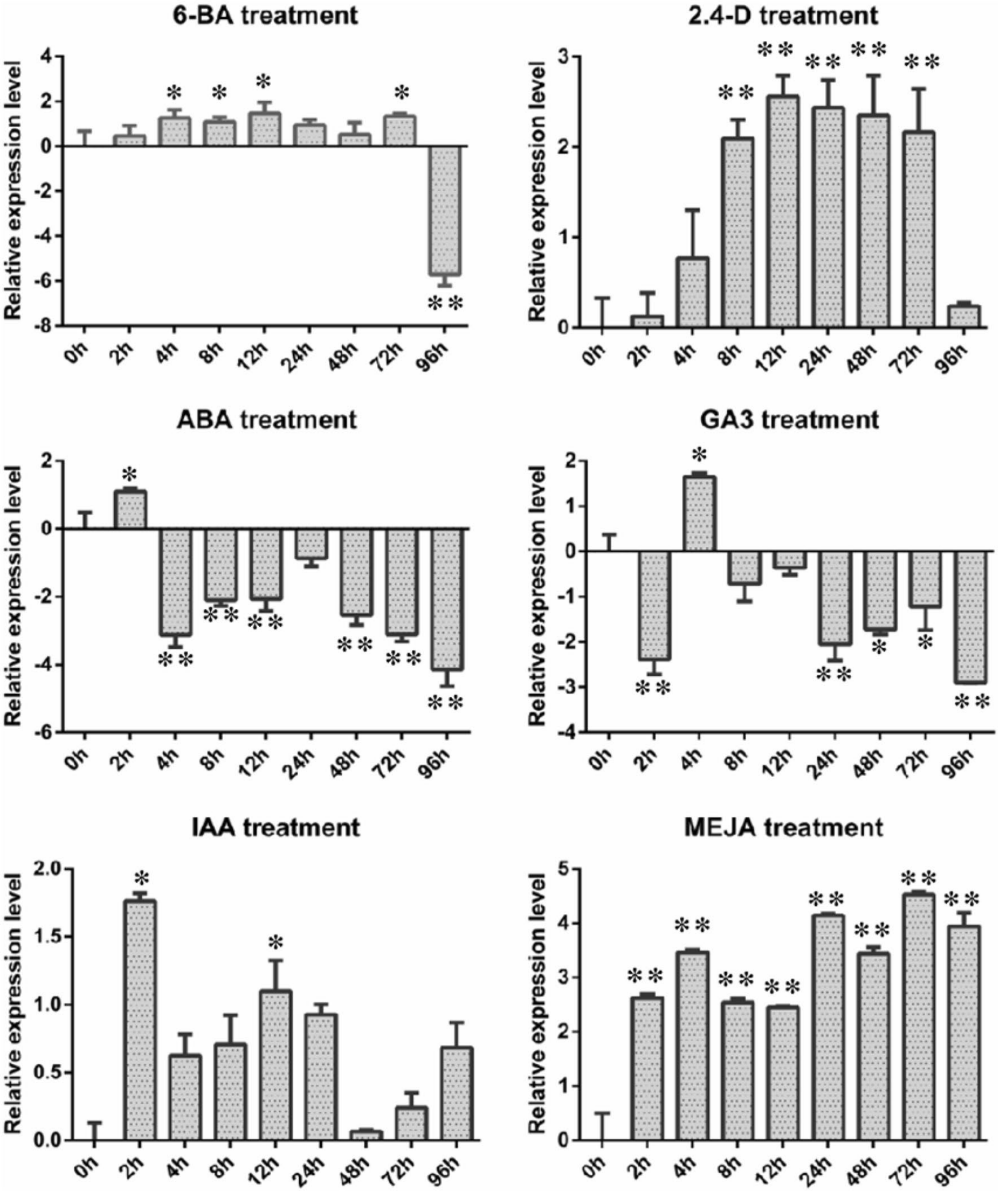
*LoHDZ2* expression levels under different hormone treatments. *Compared with 0 h treatment, the difference was significant; **Compared with 0 h treatment, the difference was extremely significant.

After *Larix olgensis* seedlings were treated with 50 mg·L^−1^ GA_3_, IAA, ABA, 6-BA, MeJA, or 2,4-D solutions or water (control), according to the qRT-PCR results(Fig. 2, and see Supplementary information), it showed that the expression of *LoHDZ2* was downregulated sixfold after treatment for 96 h with 6-BA. However, the expression was upregulated during 0–72 h of treatment. The expression of *LoHDZ2* was downregulated fivefold from 4 to 96 h after ABA treatment. The expression of *LoHDZ2* was upregulated within 96 h after IAA, 2,4-D and MeJA treatment.

### Plasmid construction

**Figure 3:**
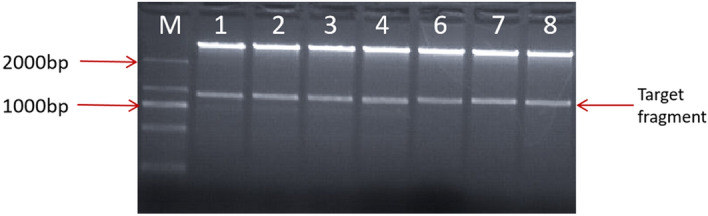
Double restriction digestion of the target gene and vector. M: 2000 bp DNA molecular marker; 1–8: transgenic lines.

**Figure 4:**
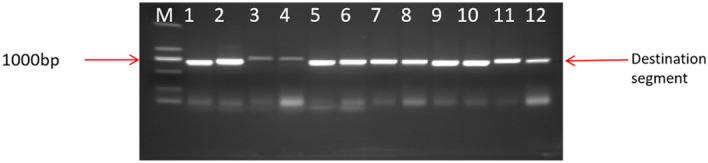
PCR detection of the target gene in *Agrobacterium*. M: 2000 bp DNA molecular marker; 1–12: transgenic lines.

The total length of the *LoHDZ2* was 945 bp; it encoded a protein with 314 amino acids and a molecular weight of 36.89 kDa^15^. *LoHDZ2* belongs to the HD-ZipII subfamily. The total RNA of *Larix olgensis* was extracted with an RNA extraction kit and reverse transcribed into cDNA. This gene was then amplified with cDNA as the template, and then the restriction enzymes XbaI and BamHI were used to double digest the pCAMBIA1302 vector (Fig. 3). After ligation with T4 ligase, the vector which was temporarily named p1302*-LoHDZ2,* was inserted into *E. coli* DH5α cells. A random colony containing the pCAMBIA1302 vector was selected for confirmation with universal and gene-specific primers via PCR. The positive plasmid sequencing results showed that *LoHDZ2* was present in the pCAMBIA1302 vector, there were no amino acid mutations, and creation of the p1302*-LoHDZ2* vector was successful (Fig. 4).

### Transient expression of GFP in tobacco by injection

The VB191104-*LoHDZ2*-eGFP vector was successfully constructed, and the OD value of Agrobacterium tumetobacter was adjusted to 0.6. The tobacco leaves were injected with the infection solution, and the GFP signal was detected by fluorescence microscope or laser co-fluorescence microscope.

**Figure 5:**
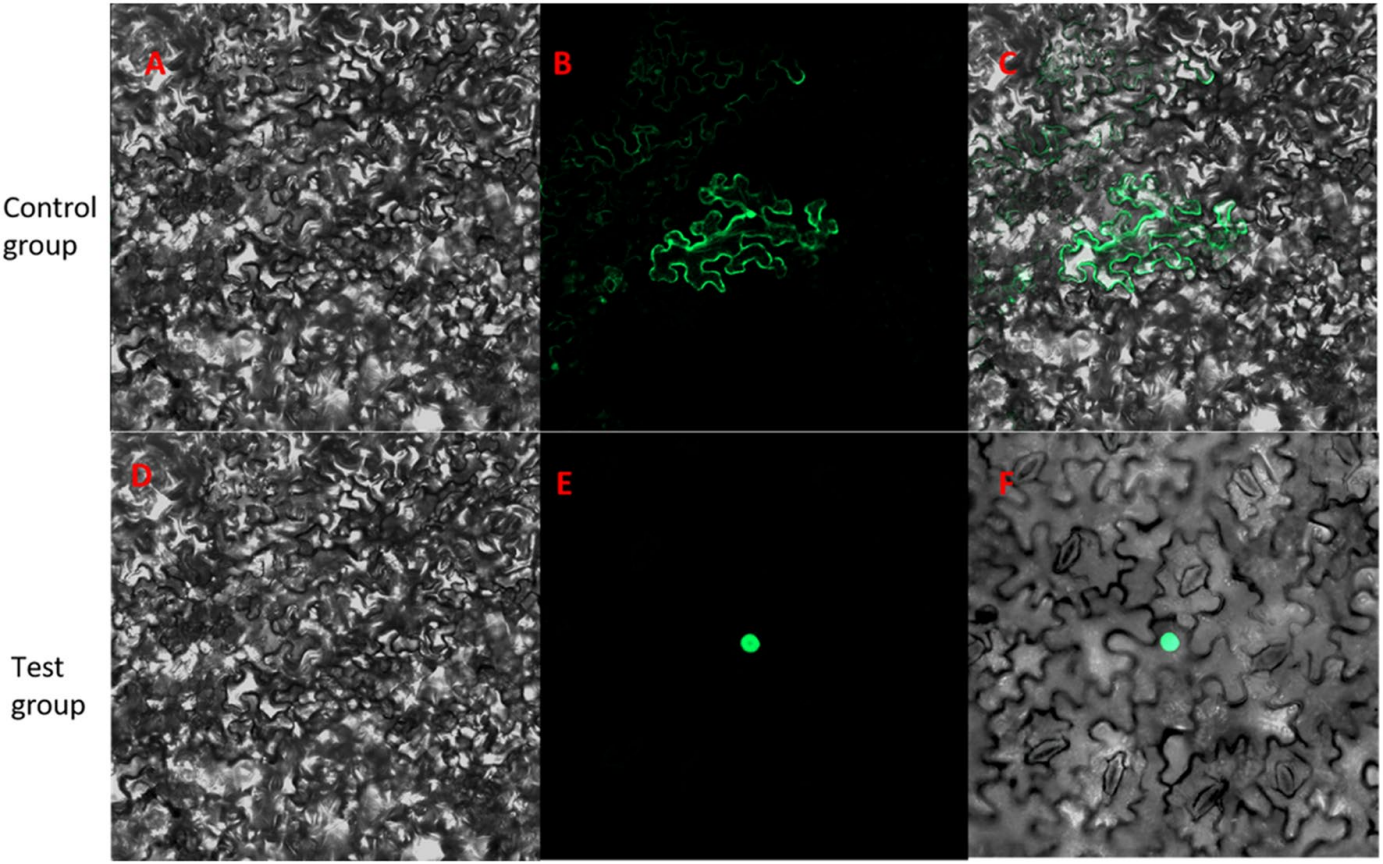
*LoHDZ2* subcellular positioning results. (**A**) D dark field observation; (**B**) E tom observation; (**C**) F is the combination of both.

Based on the results of the experiment, *LoHDZ2*-eGFP can only detect the green fluorescent signal expressed by GFP in the nucleus, while the signal of GFP in the control group can be detected in the whole cell (Fig. 5). The above experimental results indicate that *LoHDZ2*-eGFP is located in the nucleus.The subcellular localization results are consistent with the basic feature that most transcription factors are located in the nucleus.

### Antibiotic susceptibility results

**Figure 6:**
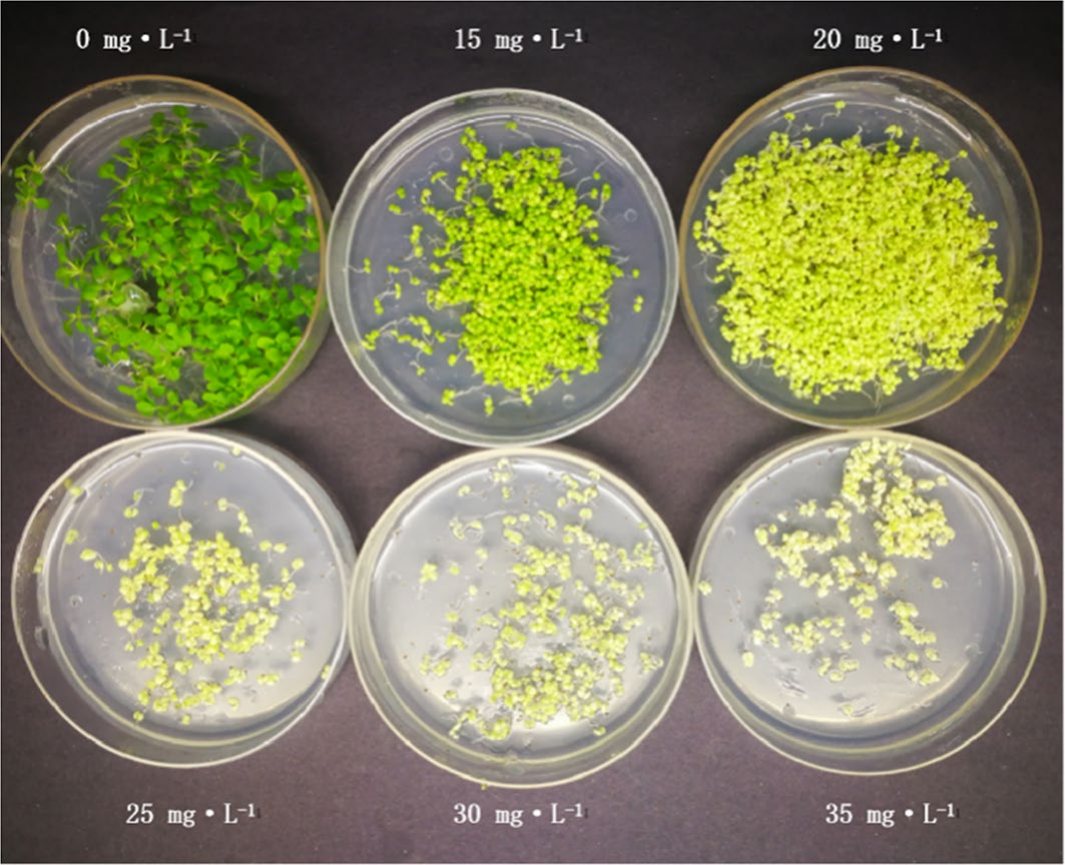
Growth status of wild-type tobacco in different concentrations of hyg.

Resistance screening was performed in culture medium with different concentrations of hyg. Hyg influenced tobacco seed germination and growth after a week of culture in medium containing different concentrations of hyg (Fig. 6). In the medium containing 25, 30 and 35 mg·L^−1^ hyg, embryonic callus growth was restrained and presented a blade-yellowing phenomenon. We found that 25 mg·L^−1^ hyg was suitable for screening tobacco genetic transformants.

### Genetic transformation and detection of the p1302-LoHDZ2 vector in tobacco

**Figure 7:**
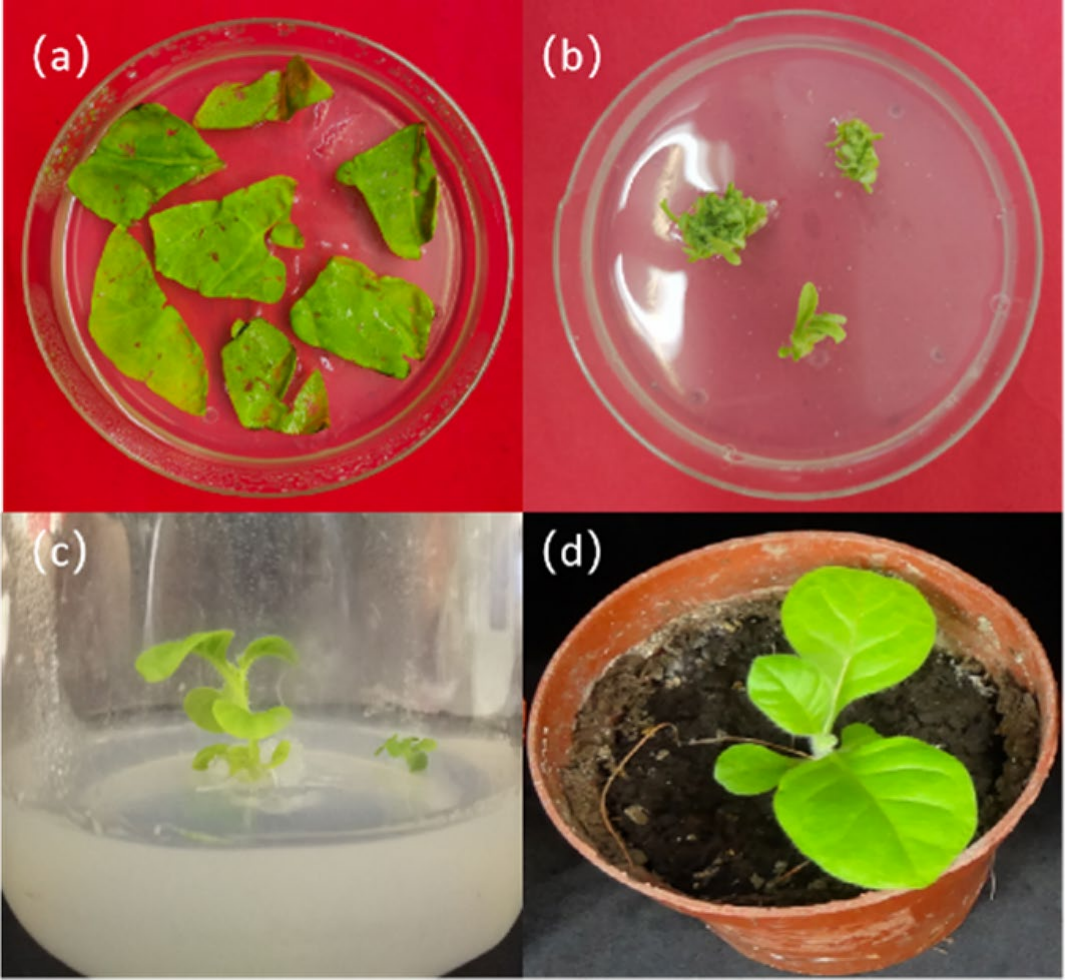
Resistance screening of genetically modified tobacco. (**a**) Cocultivation; (**b**) Induced resistance of differentiated buds; (**c**) Rooted tissue culture seedlings; (**d**) Transplantation into soil.

The leaf-disk transformation method was adopted, and *Agrobacterium tumefaciens* GV3101 (p1302*-LoHDZ2*) was used to infect tobacco leaves. The transgenic leaves were screened for resistance (Fig. 7a). After the resistant buds were isolated, they were subcultured on differentiation medium containing hyg antibiotics, and budding buds were obtained (Fig. 7b).

**Figure 8:**
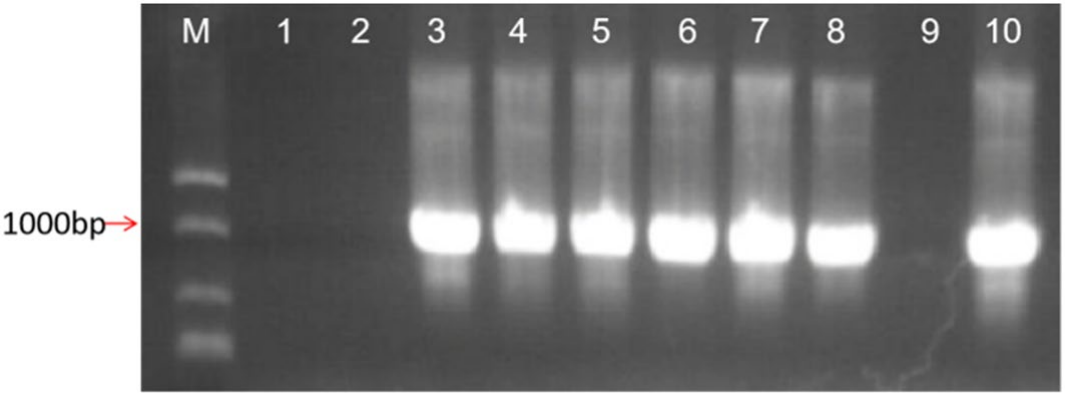
PCR detection of p1302*-LoHDZ2* in transgenic tobacco. M: 2000 bp DNA molecular marker; 1: negative control; 2: water; 3: positive control; 4–10: transgenic strains.

**Figure 9:**
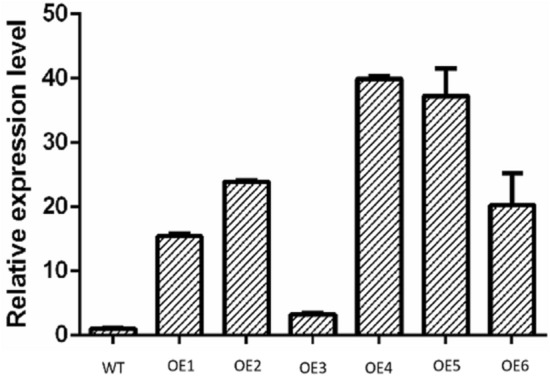
Relative expression levels of p1302*-LoHDZ2* in transgenic tobacco. WT: wild tobacco; OE2-OE5: different transgenic lines.

After the tufted buds emerged from the stems, plants were transplanting to root culture medium (Fig. 7c); after rooting, plants were transplanted to nutritional soil for cultivation(Fig. 7d).After screening for T_1_ and T_2_ generations, then cultured resistant plant; their growth phenotypes were observed. DNA was extracted from seven lines of transgenic tobacco and wild-type tobacco, and vector-specific and gene-specific primers were used for PCR detection (Fig. 8, and see Supplementary information). qRT-PCR of *LoHDZ2* was performed for four transgenic plants, and the relative expression was quantified in wild-type tobacco (Fig. 9).

The results showed that *LoHDZ2* was expressed in all four lines, and the relative expression level was high in OE2, OE4 and OE5. The three transgenic lines OE2, OE4 and OE5 were used in subsequent experiments.

### Phenotypic changes and growth indexes of transgenic tobacco

#### Phenotype determination of transgenic tobacco

**Figure 10:**
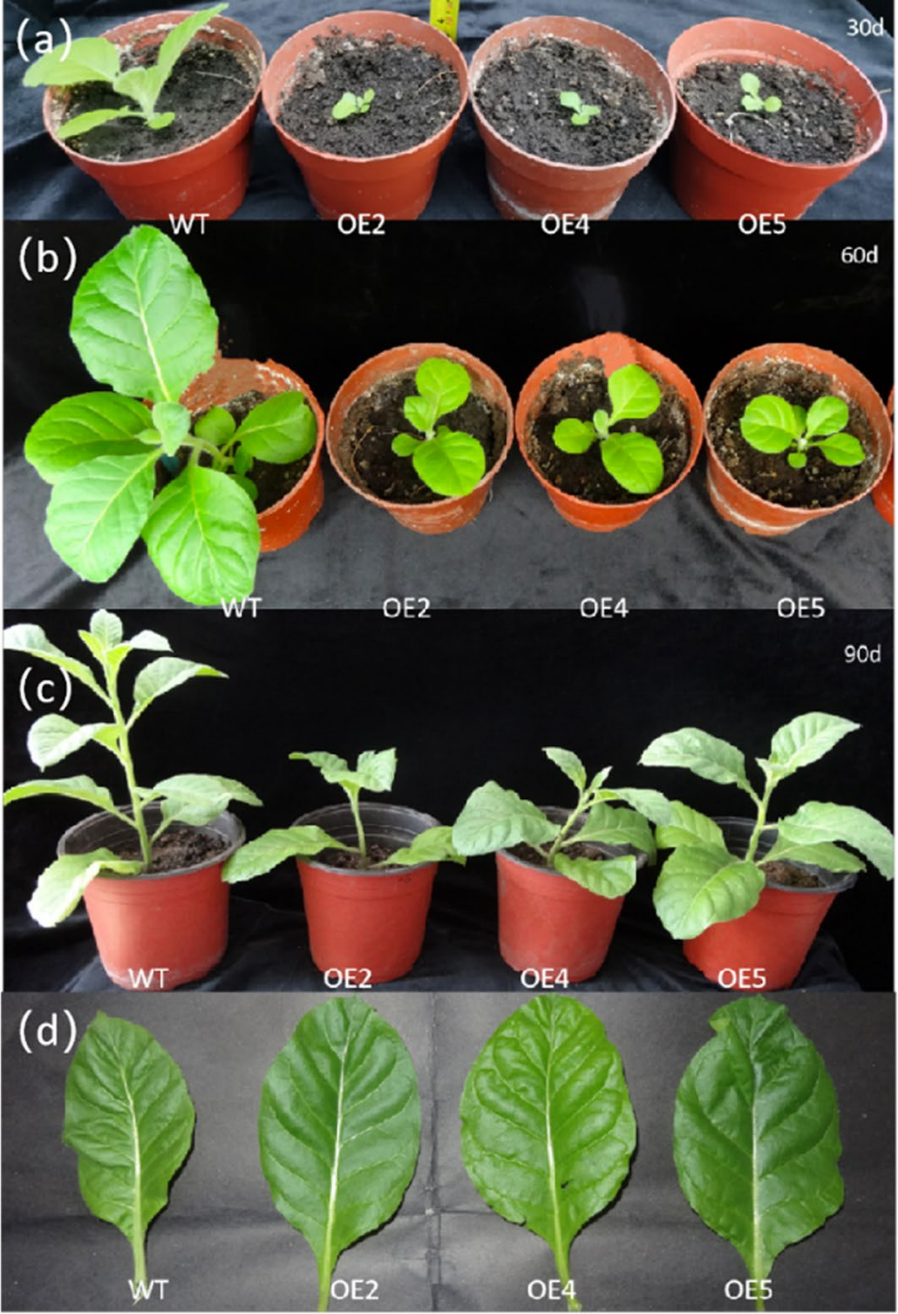
Changes in the plant height of transgenic tobacco grown for 30 days, 60 days and 90 days, as well as changes in leaf size after 90 days of growth. (**a**) Wild-type and transgenic tobacco grown for 30 days; (**b**) Wild-type and transgenic tobacco grown for 60 days; (**c**) Wild-type and transgenic tobacco grown for 90 days; (**d**) Wild-type and transgenic tobacco leaves grown for 90 days. WT: wild-type tobacco; OE2-OE5: different transgenic lines.

OE2, OE4 and OE5 were selected for character determination because of their high level of gene expression. Transgenic lines and wild-type plants were transplanted to the soil, and once plants grew true leaves, plant height was observed at different times (30 d, 60 d and 90 d) (Fig. 10). The morphological changes in wild-type tobacco after 30 d of cultivation in the greenhouse were compared with those of transgenic tobacco. We found that transgenic tobacco grew slower and shorter, demonstrating a dwarfing phenomenon. After 60 days of growth, the plant height of the three transgenic plants was shorter and the leaves were smaller than those of the wild-type tobacco seedlings. After 90 days of growth, the plant height of the three transgenic tobacco lines was still shorter than that of the wild-type tobacco, but the leaves were relatively larger.

**Figure 11:**
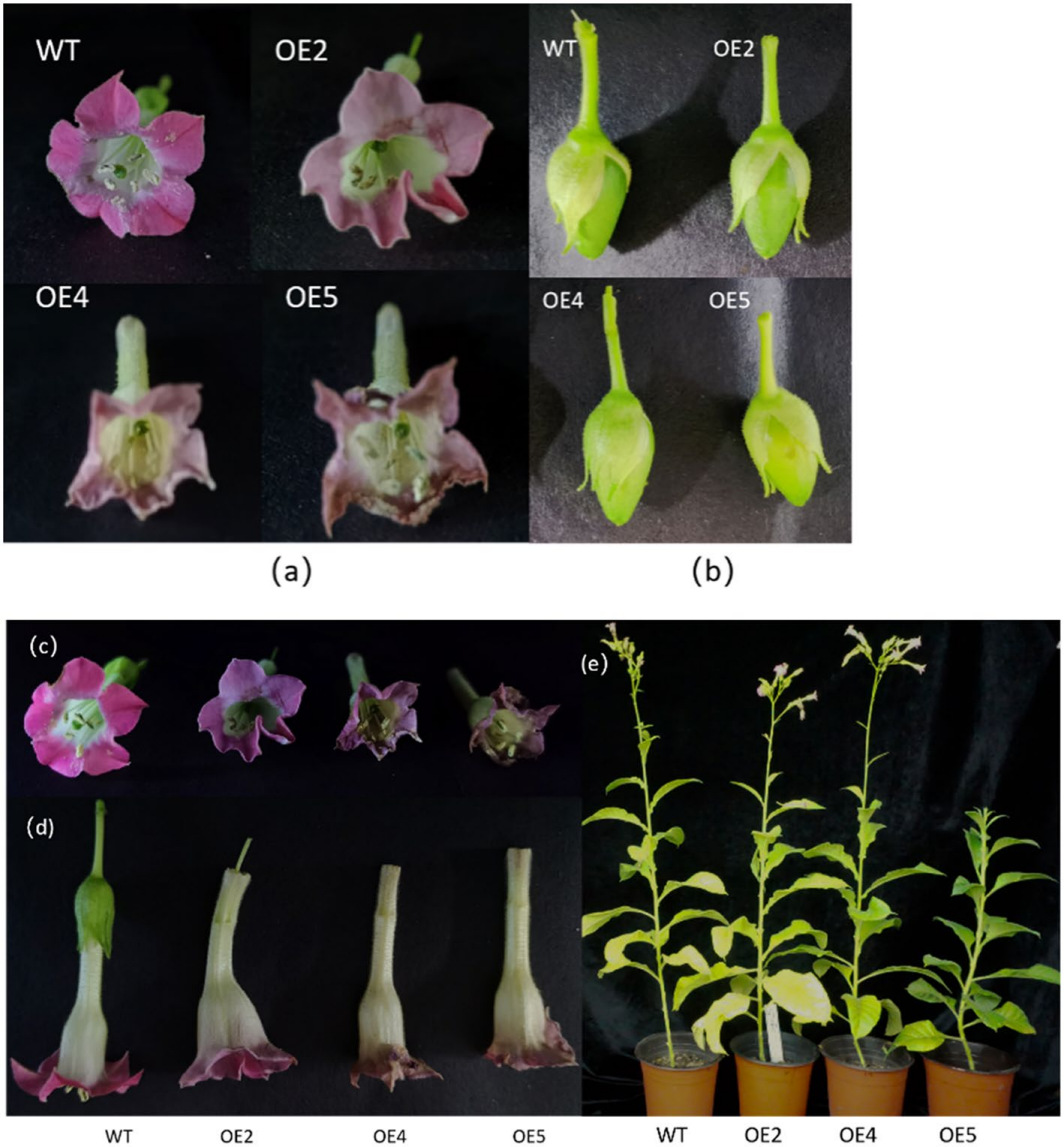
Phenotypic observation of transgenic tobacco during the reproductive period. (**a**), (**c**) Transgenic and wild-type tobacco floral organs; (**b**) Transgenic and wild-type tobacco fruits; (**d**) Unopened transgenic and wild-type tobacco flowers; (**e**) Transgenic and wild-type tobacco plants. WT: wild-type tobacco; OE2-OE5: different transgenic lines.

The transgenic plants entered the anthesis stage after 3 months of growth (Fig. 11e, and see Supplementary information), which was approximately 7–10 days earlier than wild-type tobacco. Compared with wild-type tobacco, the petals, sepals and ovary growth and color of transgenic tobacco were different; transgenic plants showed a paler corolla color, early maturity (Fig. 11a,c,d, and see Supplementary information), deeper stigma and style colors, and atrophied petals. However, the fruit development of transgenic plants was normal (Fig. 11b, and see Supplementary information), and ovules full of seeds were well developed with normal coloration in the three transgenic lines.

### Determination of the growth index of transgenic tobacco

**Figure 12:**
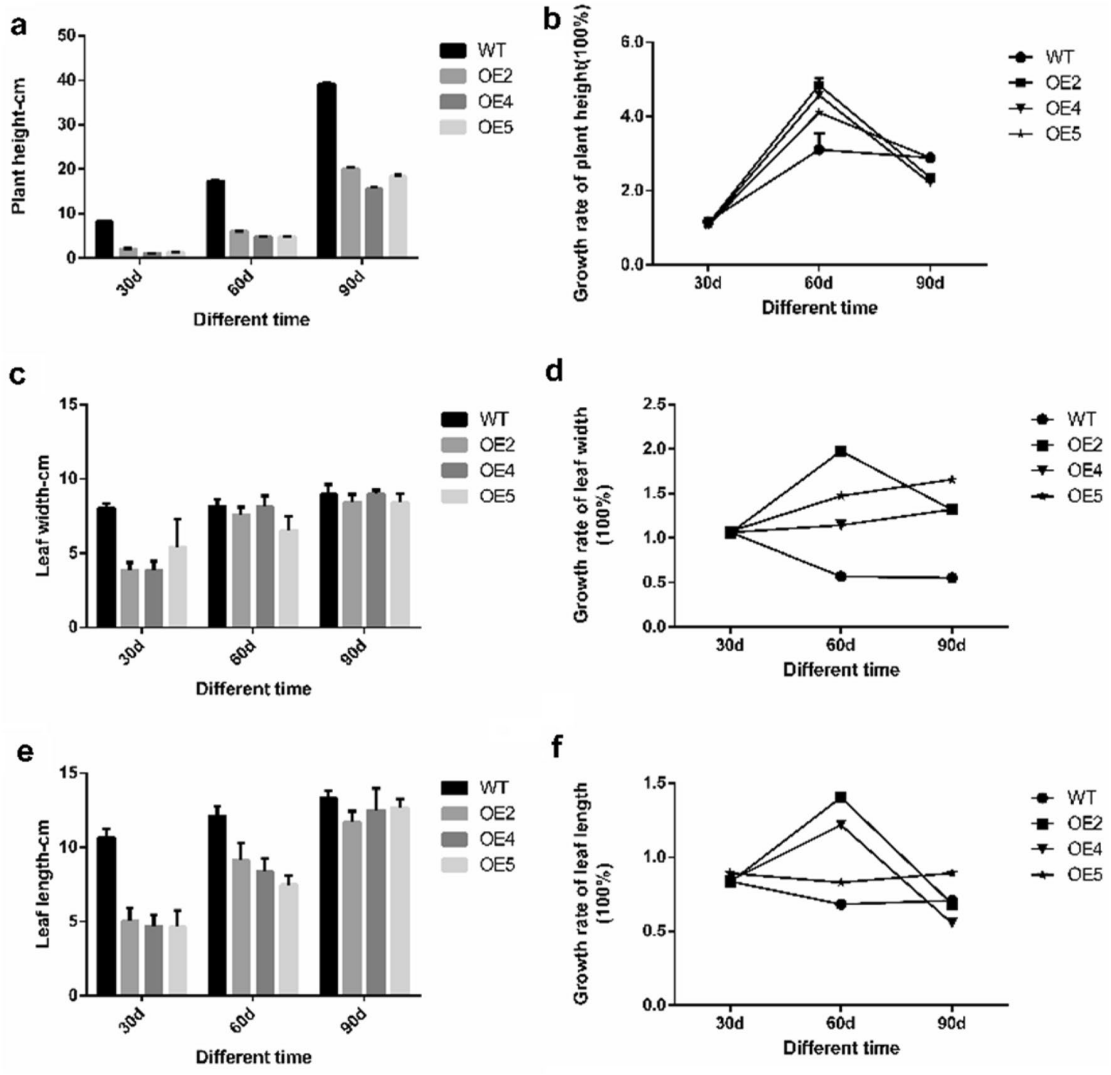
Determination of the growth index of transgenic tobacco and wild-type tobacco. (**a**) Plant height of transgenic tobacco and wild-type tobacco; (**b**) High growth rate of transgenic tobacco and wild-type tobacco; (**c**) Leaf length of transgenic tobacco and wild-type tobacco; (**d**) Leaf length change rate in transgenic tobacco and wild-type tobacco; (**e**) Leaf width of transgenic tobacco and wild-type tobacco; (**e**) Leaf width change rate of transgenic tobacco and wild-type tobacco; WT: wild tobacco; OE2-OE5: different transgenic lines.

We measured plant height, leaf length and leaf width in *LoHDZ2-OE* transgenic tobacco and wild-type tobacco grown for 30 d, 60 d and 90 d (see Supplementary information). In addition, the changes in plant high, leaf length and leaf width were calculated, and the statistical results showed that the three transgenic tobacco lines were shorter than the wild-type plants across the different growth periods; however, there was no significant difference among the three transgenic lines. The wild-type tobacco and transgenic tobacco plants from the three lines all reached their maximum growth rate in terms of plant height at 60 days (Fig. 12a). From then on, the plant growth rate slowed down. However, the growth rates of *LoHDZ2-OE2*, *LoHDZ2-OE4* and *LoHDZ2-OE5* at 60 days were higher than that of wild-type tobacco (Fig. 12b).

The results showed that the leaf width of wild-type tobacco at 30 days was signally higher than that of the three lines of transgenic tobacco. At 60 days, the leaf width of transgenic tobacco was still smaller than that of wild-type tobacco, but the difference was not significant. After 90 days of growth, the difference in the leaf widths of transgenic and wild-type tobacco was relatively small, with little difference across the three transgenic lines (Fig. 12c). The results show that the leaf growth rate of transgenic plants was higher than that of wild-type plants within 60 days of plant growth. Additionally, after 60 days of plant growth, the transgenic plants had the highest rate of leaf growth, while the wild-type tobacco had the lowest rate of leaf growth. At 90 days of plant growth, the leaf growth rate of wild-type tobacco was higher than that of transgenic plants (Fig. 12d).

After 30 days of growth, the leaf length of wild-type tobacco was obviously higher than that of transgenic tobacco. However, at 60 days and 90 days of growth, the leaf length of transgenic plants was alike to that of wild-type plants (Fig. 12e). At 60 days of growth, the leaf length growth rate of transgenic plants was significantly higher than that of wild-type plants. After 60 days, the leaf length growth rate decreased obviously (Fig. 12f).

### Relative expression levels of transgenic plants under different hormone treatments

**Figure 13:**
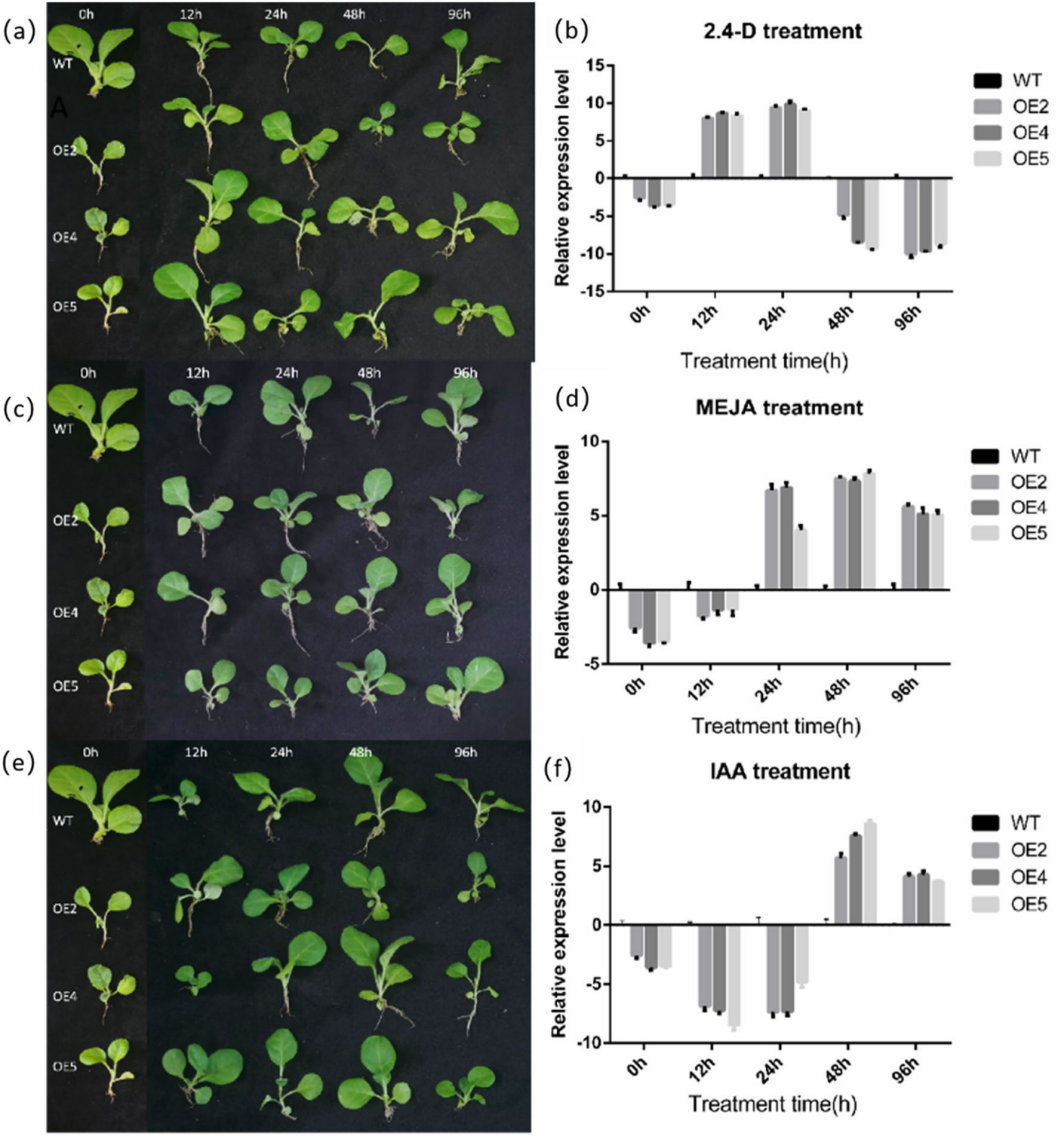
Relative expression levels of transgenic and wild-type tobacco under different hormone treatments. (**a**), (**b**) Tobacco treated with 2,4-D hormone; (**c**) ,(**d**) Tobacco treated with MeJA hormone; (**e**), (**f**) Tobacco treated with IAA hormone; *WT* wild tobacco, *OE2-OE5* different transgenic lines.

Compared to the expression of *LoHDZ2* in untreated wild-type *Larix olgensis,* the expression of *LoHDZ2* was upregulated in the plants treated with the three hormones for 96 h, so the transgenic tobacco plants were then treated with the three hormones to examine their differential gene expression. The transgenic and wild-type tobacco plants were sprayed with 50 mg · L^-1^ IAA, 2,4-D and MeJA solutions for 0 h, 12 h, 24 h, 48 h and 96 h. Then, qRT-PCR was used to determine the relative expression level under different treatments (Fig. 13).

Compared with wild-type plants, transgenic plants showed wilting 48 h after 2,4-D hormone treatment (Fig. 13a). The relative expression of *LoHDZ2* in different transgenic lines of tobacco changed over time under the 2,4-D hormone treatment. When the plants were treated with 2,4-D hormone for 0 h, the expression of *LoHDZ2* in the three transgenic lines was about 4 times lower than that of wild-type plants. After treatment for 12 h and 24 h, the expression of *LoHDZ2* in all three transgenic lines was about 7–8 times higher than that of wild-type tobacco. After treatment for 48 h and 96 h, the expression of *LoHDZ2* in three transgenic strain was 8 to 9 times lower than that in the wild-type tobacco. The data show that across all 2,4-D hormone treatments, the expression levels of *LoHDZ2* in the three transgenic lines were not significantly different (Fig. 13b).

It can be seen that the transgenic seedlings after MeJA treatment for 96 h were in good condition (Fig. 13c). After treatment with MeJA (Fig. 13d), the expression levels of transgenic plants were different from the wild-type tobacco. After treatment with MeJA for 0 h, the expression of *LoHDZ2* in the three transgenic lines was approximately 4 times lower than that in the wild-type tobacco. After 12 h of treatment, the expression of *LoHDZ2* in all three transgenic lines was approximately 3 times lower than that of wild-type tobacco. After 24 h and 48 h of MeJA treatment, the expression of *LoHDZ2* in the three transgenic lines was approximately 7–8 times lower than that of wild-type tobacco, and there was little difference between the *LoHDZ2-OE2* and *LoHDZ2-OE4* plants. After 96 h, the expression of *LoHDZ2* in the three transgenic lines was approximately 6 to 7 times higher than that in wild-type tobacco, but compared with the expression at 24 h and 48 h, the level was lower.

The transgenic seedlings grew well after IAA hormone treatment for 48 and 96 h (Fig. 13f). It can be seen that after IAA hormone treatment, the relative expression of *LoHDZ2-OE2* and *LoHDZ2-OE4* plants were differed. After treatment with IAA for 0 h, the expression of *LoHDZ2* in the three transgenic lines was approximately 4 times lower than that in the wild-type tobacco. After treatment for 12 h, the expression of *LoHDZ2* in all three transgenic lines was approximately 7–9 times lower than that of wild-type tobacco, and the expression of *LoHDZ2* in *LoHDZ2-OE5* was the lowest, which was approximately 9 times than that of wild-type tobacco. After treatment for 24 h, the expression of *LoHDZ2* in the three transgenic lines was approximately 7–8 times lower than that in the wild-type tobacco, and the expression of *LoHDZ2* in *LoHDZ2-OE4* was lower than that in *LoHDZ2-OE5*. After treatment for 48 h, the expression of *LoHDZ2* in the three transgenic lines was approximately 6–9 times higher than that in the wild-type tobacco, and the expression of *LoHDZ2* in *LoHDZ2-OE2* and *LoHDZ2-OE4* was still lower than that in *LoHDZ2-OE5*. After 96 h, the expression of *LoHDZ2* in the three transgenic lines was approximately 4–5 times higher than that of wild-type tobacco, and the expression level was lower than that after 48 h of treatment (Fig. 13f).

## Discussion

Based on the available data, no studies have reported on the function of HD-Zip family genes in *Larix olgensis*. Some Arabidopsis homeodomain leucine zipper (HD-zip) transcription factors are thought to rapidly induce responses to changing environmental conditions and integrate hormone signals^16^. The gene encoding the HD-Zip protein is highly conserved in evolution and has also been identified in basal plant species^17,18^. Another commonality among HD-Zip proteins is that their recognition of pseudopalindromic cis-elements, which has been notarized in DNA-binding studies.

The transcription HD-ZipII factors are known to play an important role in shade avoidance. Several members of the HD-ZipII transcription factors have been shown, they play an pivotal role in responses to shade^19^. In contrast to *Arabidopsis* HD-ZipI proteins composed of homologous domains fused to the leucine zipper domain, most HD-ZipII proteins contain an amphiphilic inhibitory motif associated with an amino-terminal ethylene reactive element binding factor(EAR‐domain). The EAR domain containing the protein is commonly used as a transcriptional suppressor^20^.

*LoHDZ2* belongs to the HD-ZipII transcription factor family. In previous studies, we preliminarily analyzed the expression of three genes (*LoHDZ2, LoHDZ11* and *LoHDZ13*) and found that their expression is the highest in nonlignified roots, indicating that they might be related to the growth of *Larix olgensis*^15^. First, we chose six plant hormones to treat *Larix olgensis*, and we used qRT-PCR to analyze the expression of *LoHDZ2* under different treatments of different time. We found that within 96 h of treatment with 2,4-D, IAA and MeJA, the expression of *LoHDZ2* was upregulated, it indicated that this gene was responsive to all three hormones. We hypothesized that this gene might be involved in plant growth.

Afterwards, in order to determine the function of *LoHDZ2,* we cloned *LoHDZ2* and inserted it into tobacco by genetic transformation. The phenotype of transgenic tobacco was determined, and the transgenic plants were subjected to stress and qRT-PCR detection. The results showed that the transgenic plants showed a dwarf phenotype, but the growth rate of transgenic seedlings was higher than that of wild-type tobacco.But the plant dwarfing may be affected by the CaMV35S promoter in the vector, because low expression or silencing of genes driven by CaMV35S promoter was found in chrysanthemum^21^. However, it is also possible that HD-ZipII subfamily transcription factor genes inhibit the expression of a downstream regulatory gene. Studies have shown that the interacting transcription factors in this family have been duplicated multiple times to modulate gene expression across diverse processes, including hormone signaling, stress responses, and the control of flowering time, for which we also found similar results^22^.

Additionally, at 60 days of plant growth, both the seedling height and leaf growth rate of transgenic plants reached the maximum. The transgenic plants bloomed a week earlier than the wild-type plants, but the growth and ripening of seeds was not affected. It can be seen that transgenic plants might put more energy into reproductive growth rather than vegetative growth. According to existing studies, Ciarbelli found that induction of HD-ZipII and increased levels of the plant hormone auxin seem to be essential for complete shade avoidance^23^, which was similar to our results.

Plant growth and development are regulated by nutrient endogenous hormones and the growth environment. Plant hormones are involved in almost every process regulating plant growth and development. Plants must adapt to the external environment in order to survive. After IAA and 2,4-D treatment, *LoHDZ2* showed higher expression within 12 to 24 h, but after 48 h of treatment, it showed lower expression. As is known, growth hormones within a certain concentration range can promote plant growth, but beyond this range, these hormones can inhibit the growth of plants. It was found that the auxin synthesis and transport of hat3 and athb4 double mutants were affected^24^ REV transcription induces genes encoding growth protein biosynthetase^25^_,_ it points to signal integration at the level of hormone signaling. The results of this study suggested that *LoHDZ2* may be involved in the regulation of metabolism, as well as the transport and regulation of plant growth hormones, which further suggested that *LoHDZ2* may be involved in promoting plant reproductive growth and inhibiting plant vegetative growth.

MeJA plays an crucial role in the regulation of plant growth and development. MeJA can inhibit root growth^26^, seed germination and tuber induction^27^, the formation of trichomes, and the development of flowers, pollen sacs, and pistils, as well as pollen sac cleavage and filament elongation^28^. MeJA plays an important part in the stress response of plants. After 24 h of treatment with MeJA hormone the expression of *LoHDZ2* was significantly upregulated, but the plant wilting rate was faster than that of wild-type tobacco. Therefore, it was speculated that *LoHDZ2* might inhibit the expression of downstream stress-related genes. The specific function of *LoHDZ2* still needs to be further studied. Our research on the function of *LoHDZ2* is still ongoing.

## Supplementary Information

Supplementary Information.

## Data Availability

All data is the original data of the experiment, and the original drawing data is contained in a separate folder.

## Abbreviations

2,4-D: 2,4-Dichlorophenoxyacetic acid
6-BA: 6-Benzylaminopurinehydrochloride
ABA: Applied behavior analysis
Bp: Base pair(s)
cDNA: DNA complementary to RNA
d: Day
dNTP: Deoxyribonucleoside triphosphate
GA_3_: Gibberellin
Hyg: Hygromycin
IAA: Indole-3-acetic acid
kDa: Kilodalton(s)
IL: Interleukin
LB: Luria–Bertani (medium)
Lo: *Larix olgensis*
MeJA: Jasmonic acid methylester
MS: MS Medium
Min: Minutes
OE: Over expression
qRT-PCR: RealtimeQuantitativePCR
