# Supplementary figures and images for: Genetic transformation and growth index determination of the Larix olgensis LoHDZ2 transcription factor gene in tobacco
Source: Sci Rep. 2021 Oct 20;11:20746. doi: 10.1038/s41598-021-99533-0 (PMC8528859; doi:10.1038/s41598-021-99533-0)

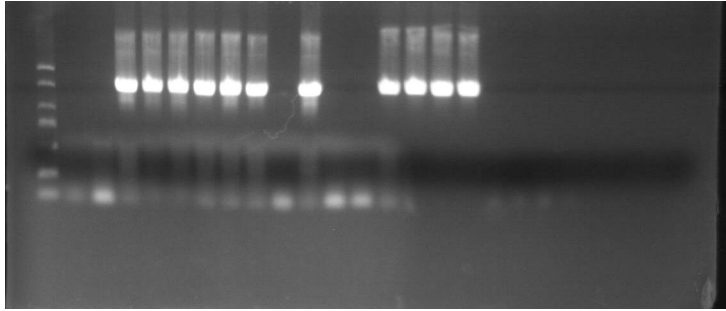

Original image of Fig. 3

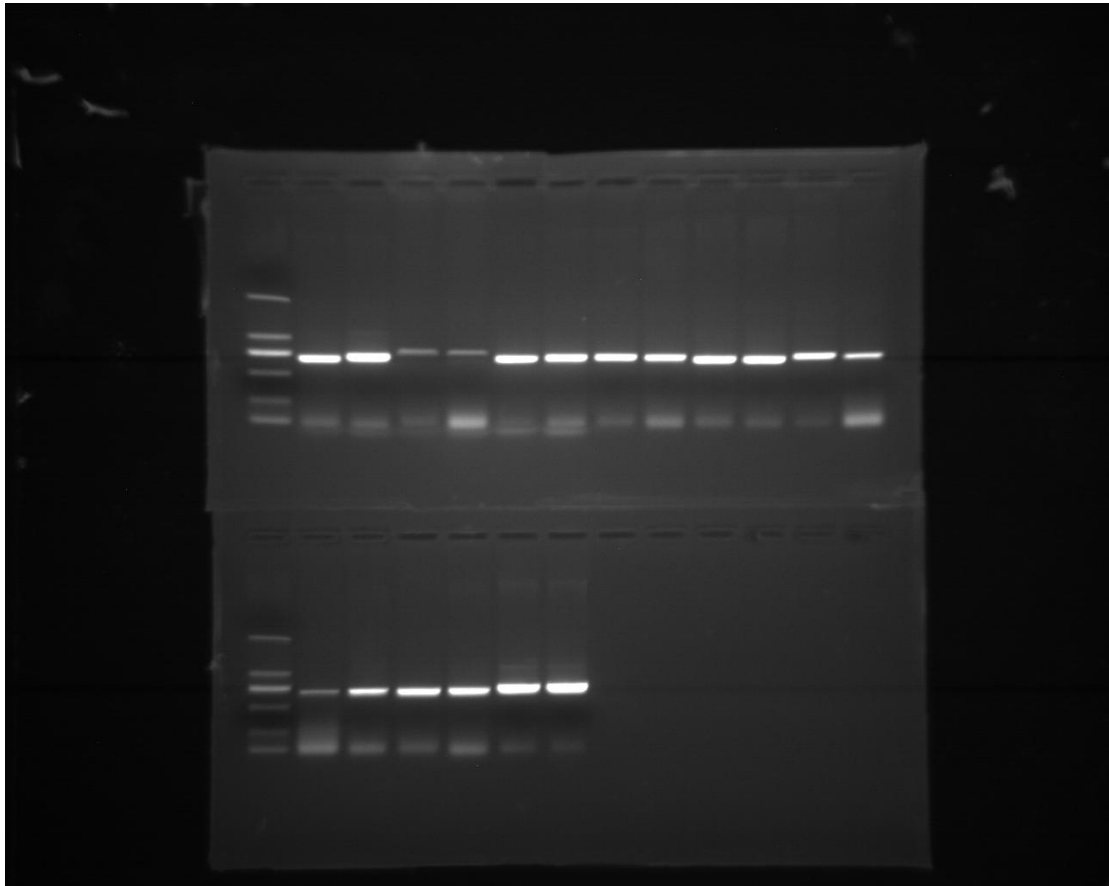

Original image of Fig. 4

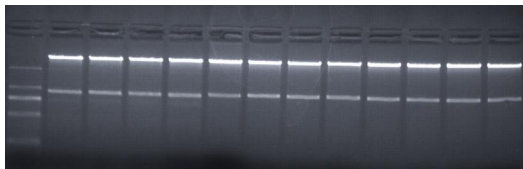

Original image of Fig. 7

Supplement: Supplementary file 1 — Supplementary Information. [file 41598_2021_99533_MOESM1_ESM.pdf]
